# Supplementary material for: miR-23b-3p Modulating Cytoprotective Autophagy and Glutamine Addiction in Sorafenib Resistant HepG2, a Hepatocellular Carcinoma Cell Line
Source: Genes (Basel). 2022 Aug 1;13(8):1375. doi: 10.3390/genes13081375 (PMC9407556; doi:10.3390/genes13081375)
Supplement: Supplementary file 1 [file genes-13-01375-s001.zip › genes-1797213-supplementary.pdf]

(A)

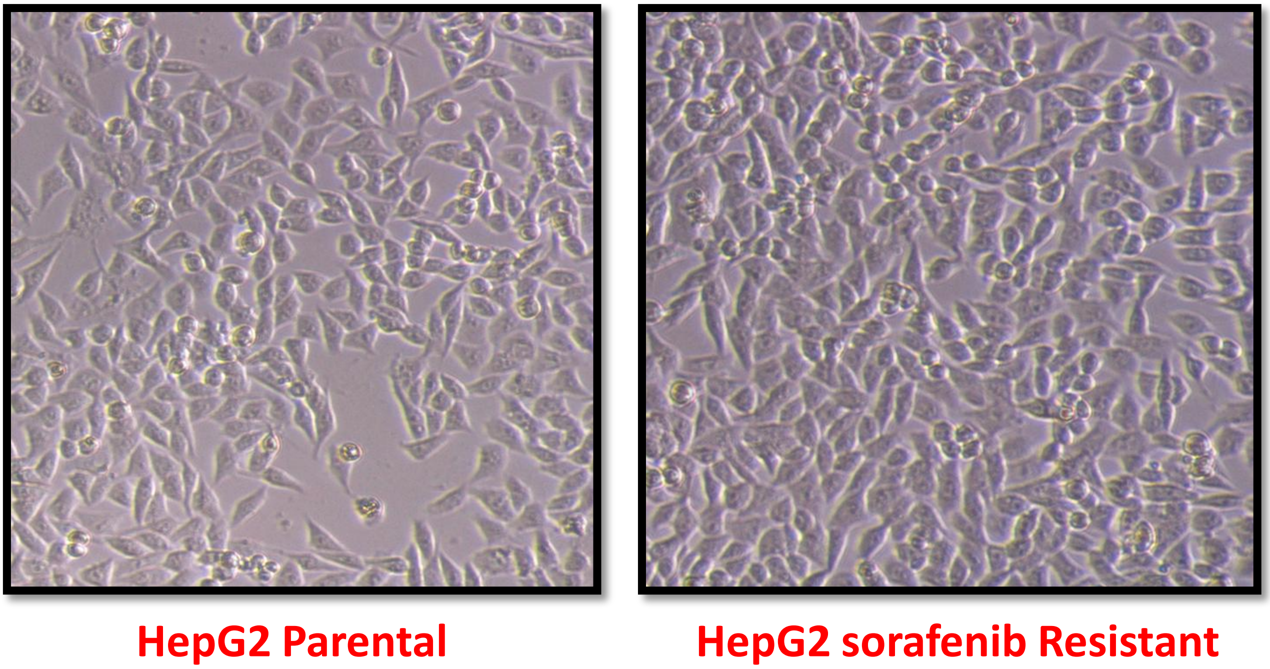

(B)

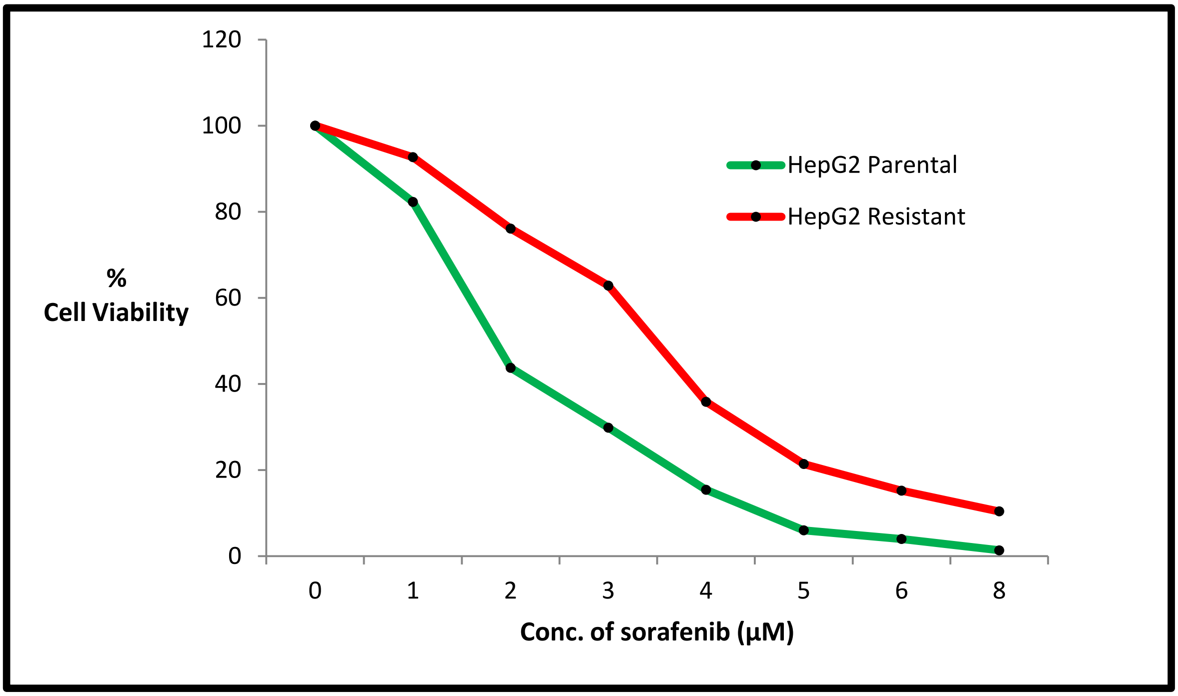

Supp. Figure S1 (A) & (B)

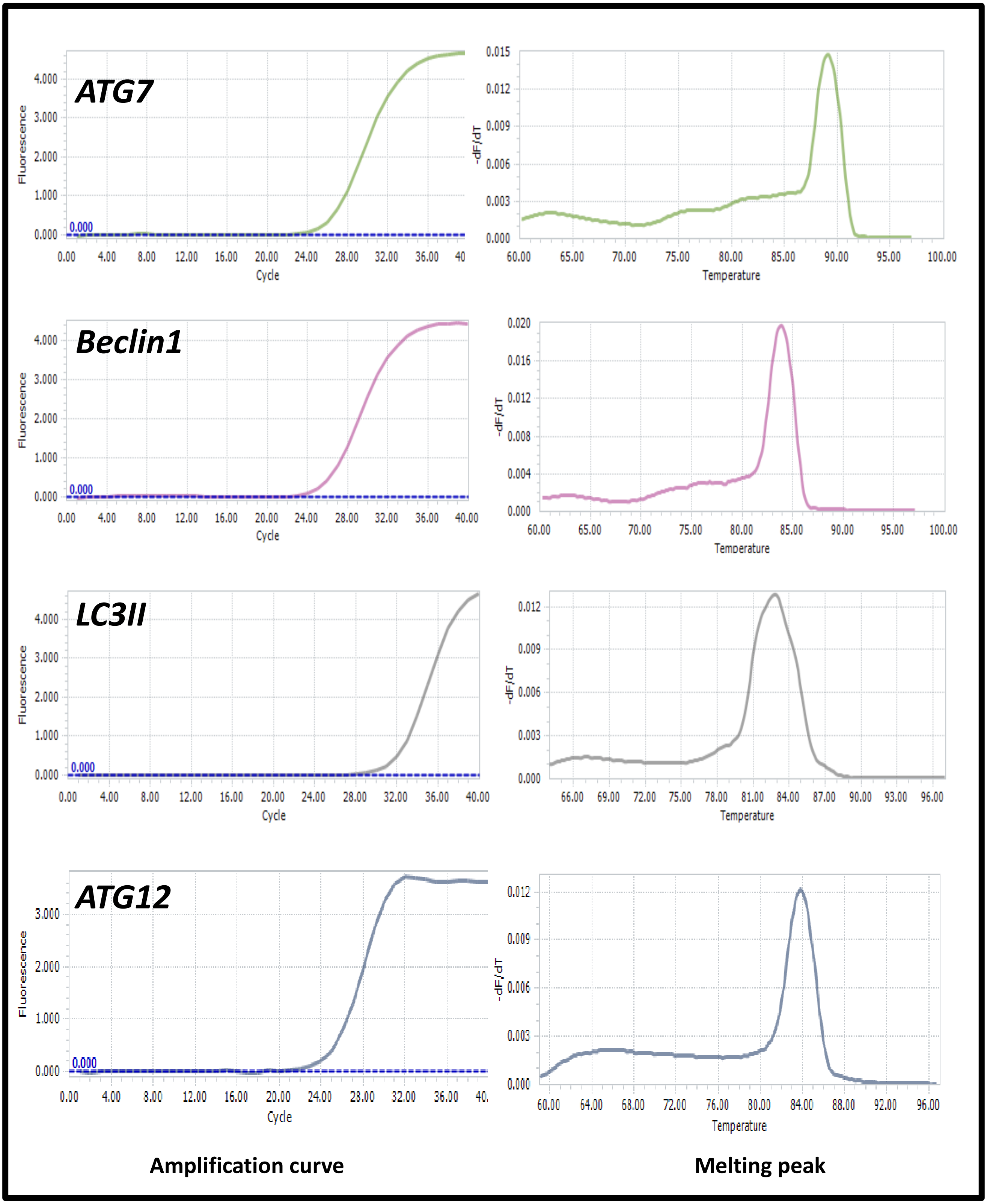

Supp. Figure S2

(A)

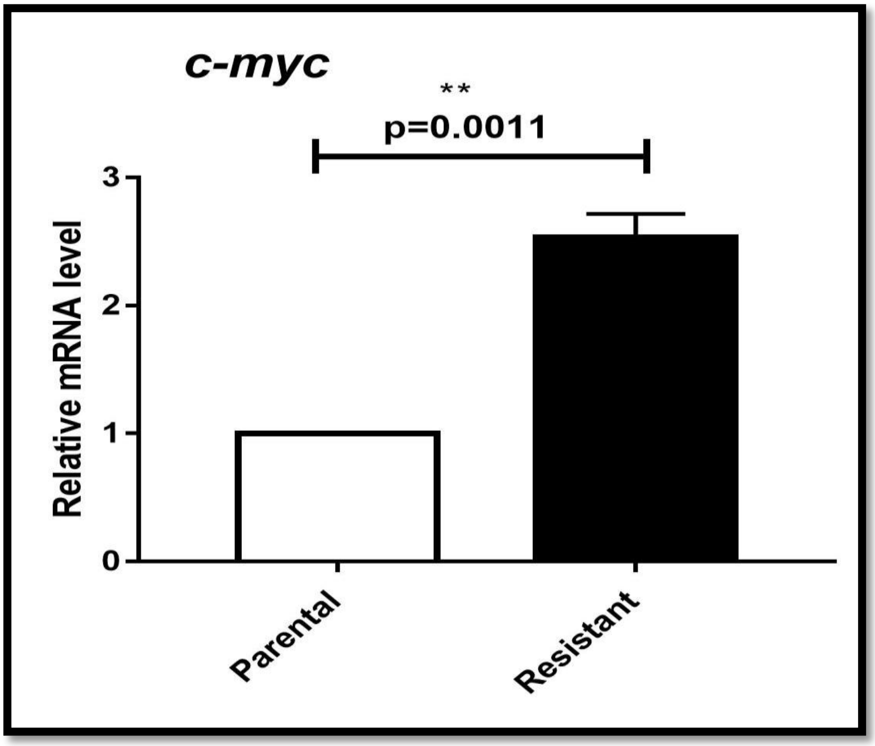

(B)

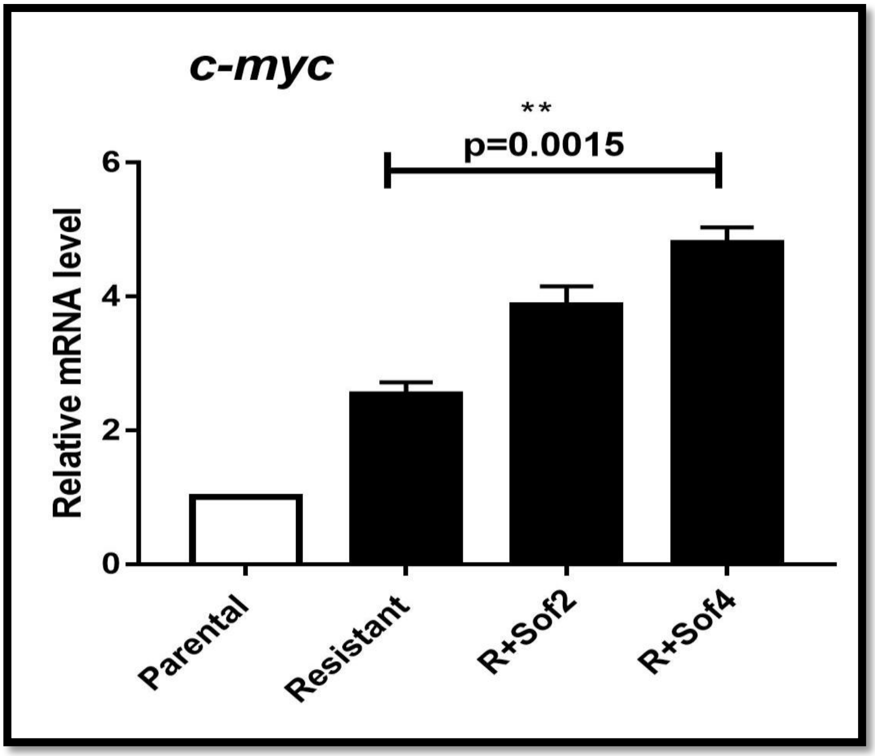

Supp. Figure S3 (A) & (B)

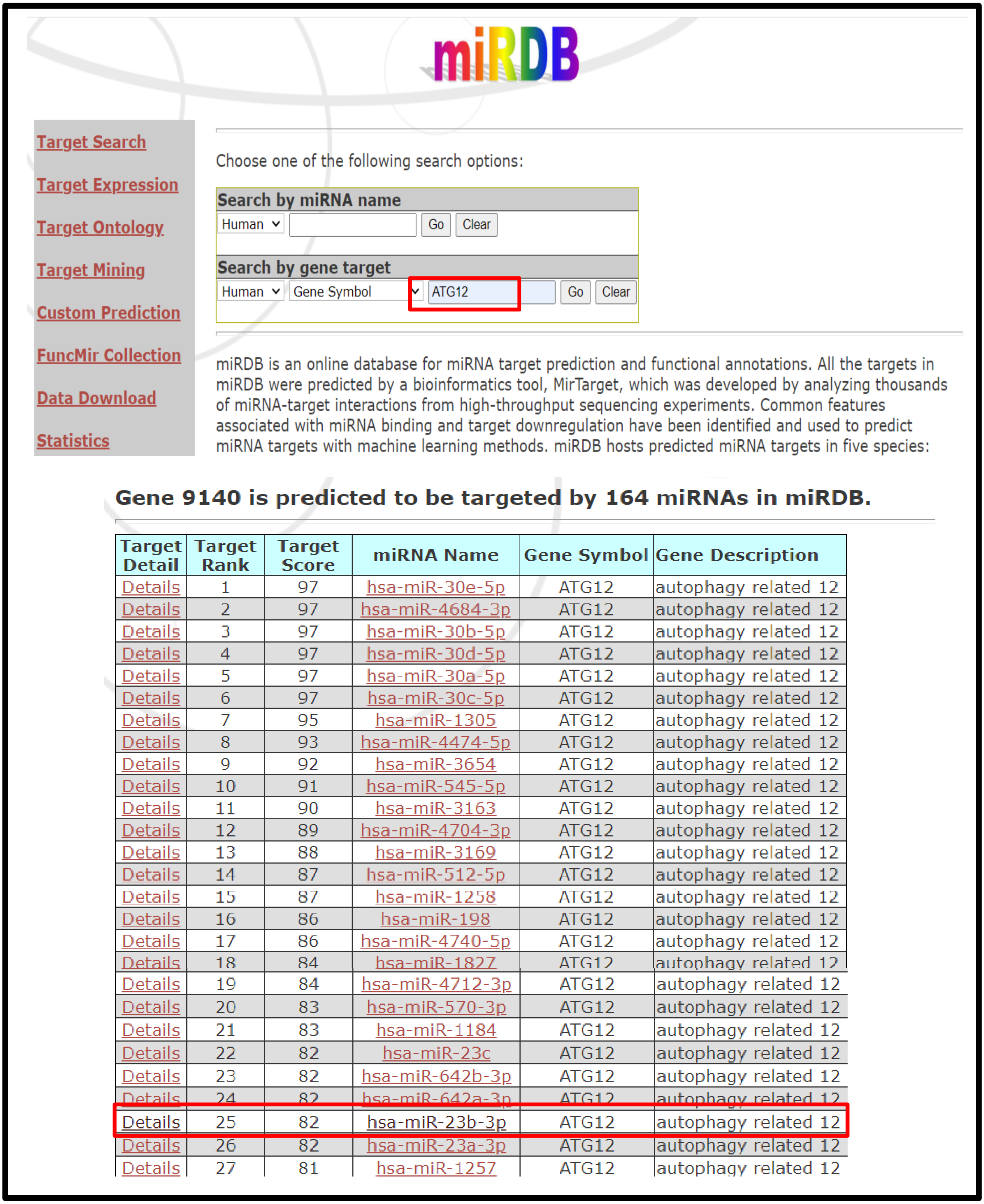

miRDB

Target Search

Target Expression

Target Ontology

Target Mining

Custom Prediction

FuncMir Collection

Data Download

Statistics

Choose one of the following search options:

Search by miRNA name

Human | Go | Clear

Search by gene target

Human | Gene Symbol | ATG12 | Go | Clear

miRDB is an online database for miRNA target prediction and functional annotations. All the targets in miRDB were predicted by a bioinformatics tool, MirTarget, which was developed by analyzing thousands of miRNA-target interactions from high-throughput sequencing experiments. Common features associated with miRNA binding and target downregulation have been identified and used to predict miRNA targets with machine learning methods. miRDB hosts predicted miRNA targets in five species:

Gene 9140 is predicted to be targeted by 164 miRNAs in miRDB.

Target Detail	Target Rank	Target Score	miRNA Name	Gene Symbol	Gene Description
Details	1	97	hsa-miR-30e-5p	ATG12	autophagy related 12
Details	2	97	hsa-miR-4684-3p	ATG12	autophagy related 12
Details	3	97	hsa-miR-30b-5p	ATG12	autophagy related 12
Details	4	97	hsa-miR-30d-5p	ATG12	autophagy related 12
Details	5	97	hsa-miR-30a-5p	ATG12	autophagy related 12
Details	6	97	hsa-miR-30c-5p	ATG12	autophagy related 12
Details	7	95	hsa-miR-1305	ATG12	autophagy related 12
Details	8	93	hsa-miR-4474-5p	ATG12	autophagy related 12
Details	9	92	hsa-miR-3654	ATG12	autophagy related 12
Details	10	91	hsa-miR-545-5p	ATG12	autophagy related 12
Details	11	90	hsa-miR-3163	ATG12	autophagy related 12
Details	12	89	hsa-miR-4704-3p	ATG12	autophagy related 12
Details	13	88	hsa-miR-3169	ATG12	autophagy related 12
Details	14	87	hsa-miR-512-5p	ATG12	autophagy related 12
Details	15	87	hsa-miR-1258	ATG12	autophagy related 12
Details	16	86	hsa-miR-198	ATG12	autophagy related 12
Details	17	86	hsa-miR-4740-5p	ATG12	autophagy related 12
Details	18	84	hsa-miR-1827	ATG12	autophagy related 12
Details	19	84	hsa-miR-4712-3p	ATG12	autophagy related 12
Details	20	83	hsa-miR-570-3p	ATG12	autophagy related 12
Details	21	83	hsa-miR-1184	ATG12	autophagy related 12
Details	22	82	hsa-miR-23c	ATG12	autophagy related 12
Details	23	82	hsa-miR-642b-3p	ATG12	autophagy related 12
Details	24	82	hsa-miR-642a-3p	ATG12	autophagy related 12
Details	25	82	hsa-miR-23b-3p	ATG12	autophagy related 12
Details	26	82	hsa-miR-23a-3p	ATG12	autophagy related 12
Details	27	81	hsa-miR-1257	ATG12	autophagy related 12

Supp. Figure S4

miRDB

- Target Search
- Target Expression
- Target Ontology
- Target Mining
- Custom Prediction
- FuncMir Collection
- Data Download
- Statistics

Choose one of the following search options:

Search by miRNA name

Human | | Go | Clear

Search by gene target

Human | Gene Symbol | GLS | Go | Clear

miRDB is an online database for miRNA target prediction and functional annotations. All the targets in miRDB were predicted by a bioinformatics tool, MirTarget, which was developed by analyzing thousands of miRNA-target interactions from high-throughput sequencing experiments. Common features associated with miRNA binding and target downregulation have been identified and used to predict miRNA targets with machine learning methods. miRDB hosts predicted miRNA targets in five species: [illegible]

Gene 2744 is predicted to be targeted by 267 miRNAs in miRDB.

Target Detail	Target Rank	Target Score	miRNA Name	Gene Symbol	Gene Description
Details	1	99	hsa-miR-4262	GLS	glutaminase
Details	2	99	hsa-miR-181b-5p	GLS	glutaminase
Details	3	99	hsa-miR-181a-5p	GLS	glutaminase
Details	4	98	hsa-miR-181d-5p	GLS	glutaminase
Details	5	98	hsa-miR-4999-3p	GLS	glutaminase
Details	6	98	hsa-miR-5696	GLS	glutaminase
Details	7	98	hsa-miR-181c-5p	GLS	glutaminase
Details	8	98	hsa-miR-3163	GLS	glutaminase
Details	9	97	hsa-miR-4803	GLS	glutaminase
Details	10	97	hsa-miR-3692-3p	GLS	glutaminase
Details	11	96	hsa-miR-548aw	GLS	glutaminase
Details	12	96	hsa-let-7b-3p	GLS	glutaminase
Details	13	96	hsa-miR-1178-3p	GLS	glutaminase
Details	14	96	hsa-miR-98-3p	GLS	glutaminase
Details	15	96	hsa-let-7f-1-3p	GLS	glutaminase
Details	16	96	hsa-let-7a-3p	GLS	glutaminase
Details	17	95	hsa-miR-200a-3p	GLS	glutaminase
Details	18	95	hsa-miR-141-3p	GLS	glutaminase
Details	39	90	hsa-miR-4482-3p	GLS	glutaminase
Details	40	90	hsa-miR-23b-3p	GLS	glutaminase
Details	41	90	hsa-miR-376c-5p	GLS	glutaminase
Details	42	90	hsa-miR-376b-5p	GLS	glutaminase
Details	43	90	hsa-miR-23a-3p	GLS	glutaminase

Supp. Figure S5

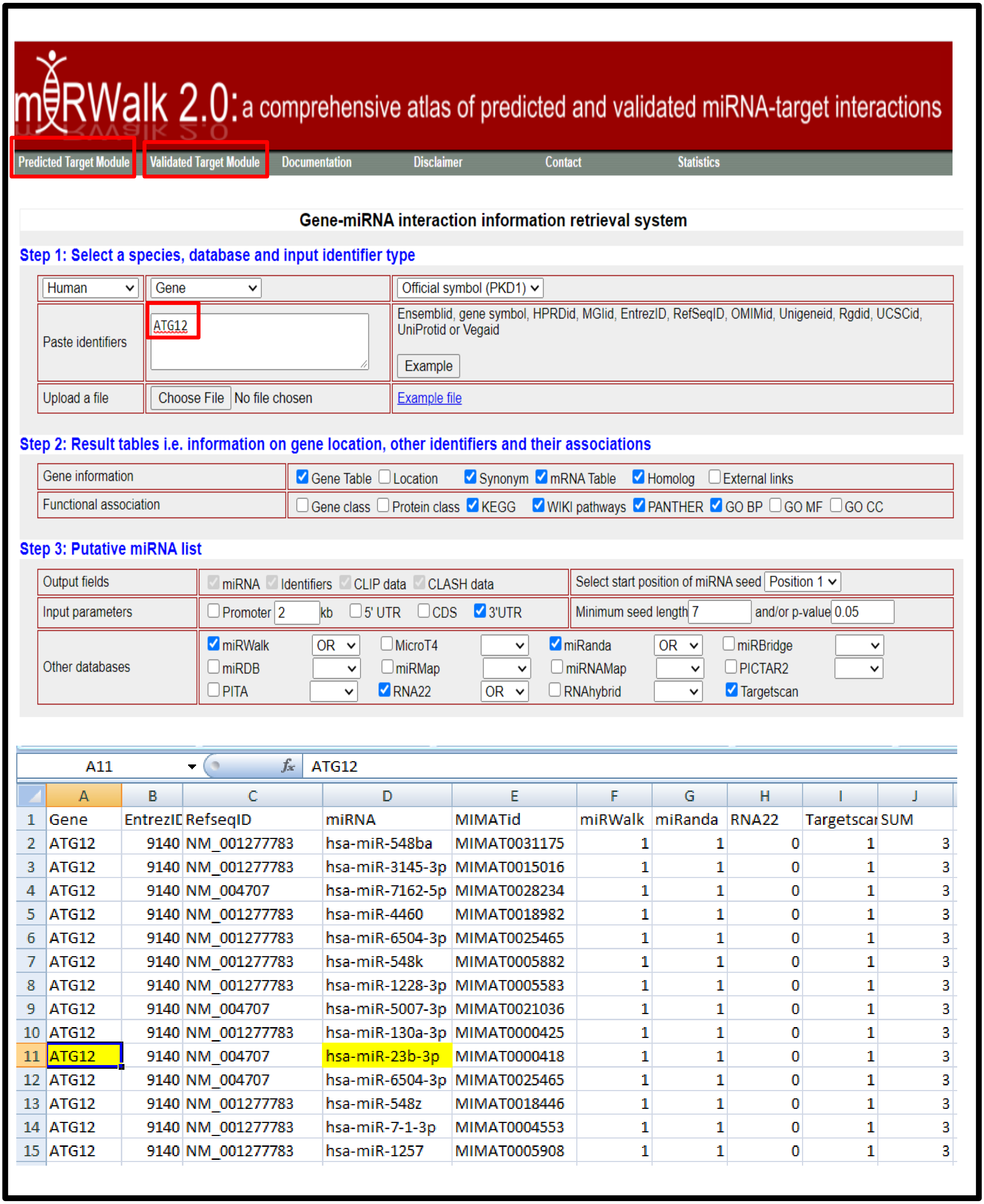

	A	B	C	D	E	F	G	H	I	J
1	Gene	EntrezID	RefseqID	miRNA	MIMATid	miRWalk	miRanda	RNA22	Targetscan	SUM
2	ATG12	9140	NM_001277783	hsa-miR-548ba	MIMAT0031175	1	1	0	1	3
3	ATG12	9140	NM_001277783	hsa-miR-3145-3p	MIMAT0015016	1	1	0	1	3
4	ATG12	9140	NM_004707	hsa-miR-7162-5p	MIMAT0028234	1	1	0	1	3
5	ATG12	9140	NM_001277783	hsa-miR-4460	MIMAT0018982	1	1	0	1	3
6	ATG12	9140	NM_001277783	hsa-miR-6504-3p	MIMAT0025465	1	1	0	1	3
7	ATG12	9140	NM_001277783	hsa-miR-548k	MIMAT0005882	1	1	0	1	3
8	ATG12	9140	NM_001277783	hsa-miR-1228-3p	MIMAT0005583	1	1	0	1	3
9	ATG12	9140	NM_004707	hsa-miR-5007-3p	MIMAT0021036	1	1	0	1	3
10	ATG12	9140	NM_001277783	hsa-miR-130a-3p	MIMAT0000425	1	1	0	1	3
11	ATG12	9140	NM_004707	hsa-miR-23b-3p	MIMAT0000418	1	1	0	1	3
12	ATG12	9140	NM_004707	hsa-miR-6504-3p	MIMAT0025465	1	1	0	1	3
13	ATG12	9140	NM_001277783	hsa-miR-548z	MIMAT0018446	1	1	0	1	3
14	ATG12	9140	NM_001277783	hsa-miR-7-1-3p	MIMAT0004553	1	1	0	1	3
15	ATG12	9140	NM_001277783	hsa-miR-1257	MIMAT0005908	1	1	0	1	3

Supp. Figure S6

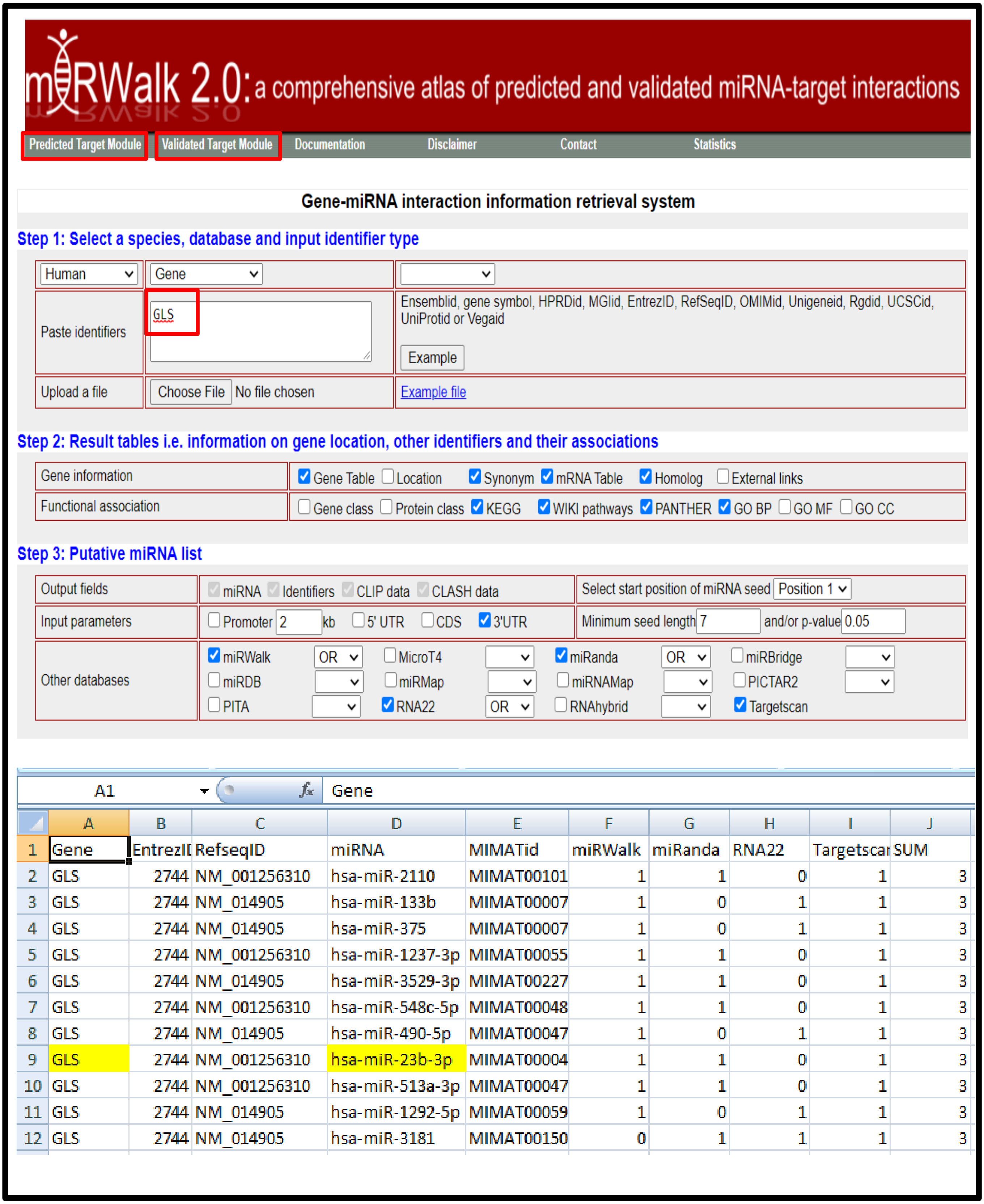

Supp. Figure S7

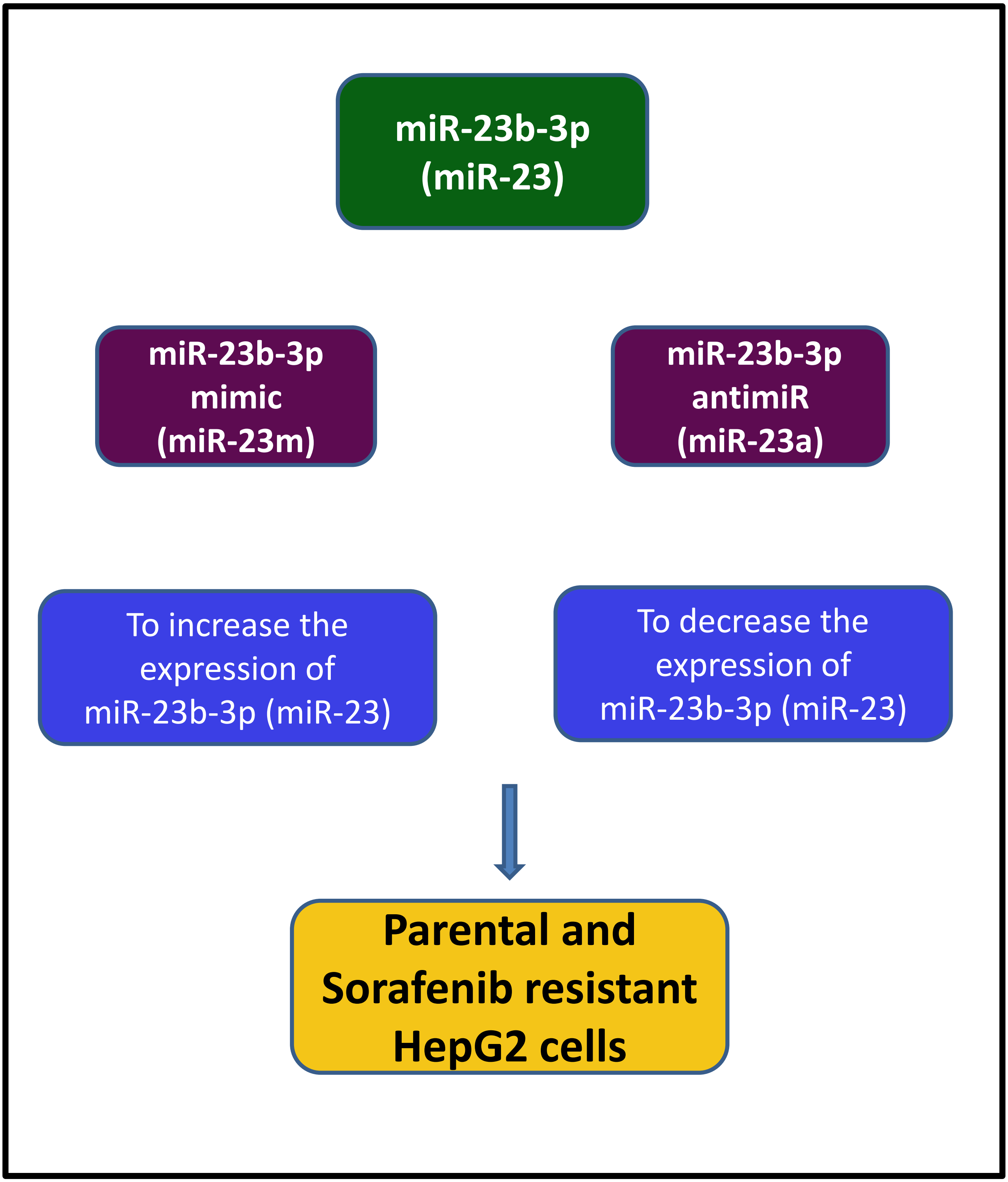

Supp. Figure S8

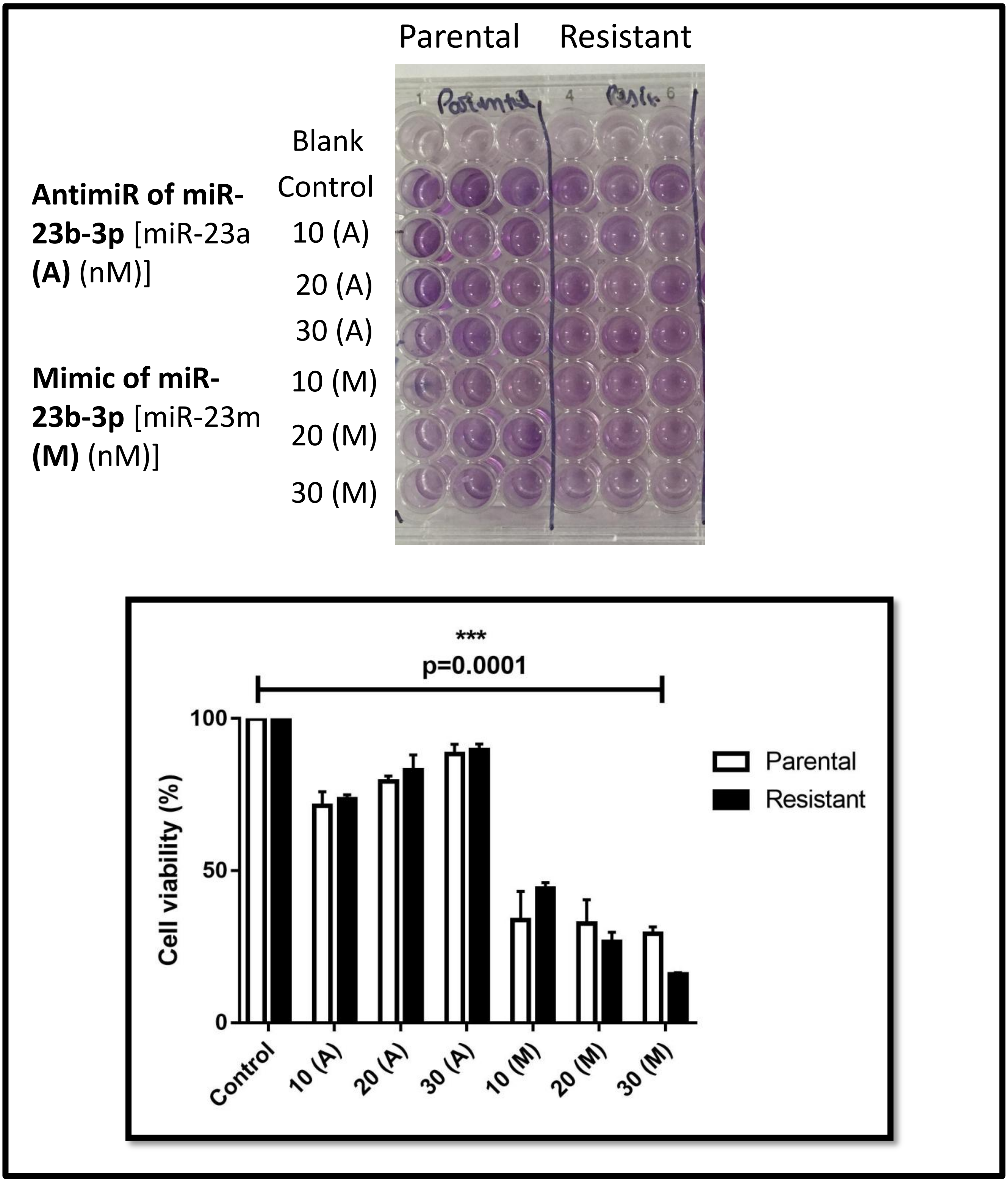

Supp. Figure S9

(A)

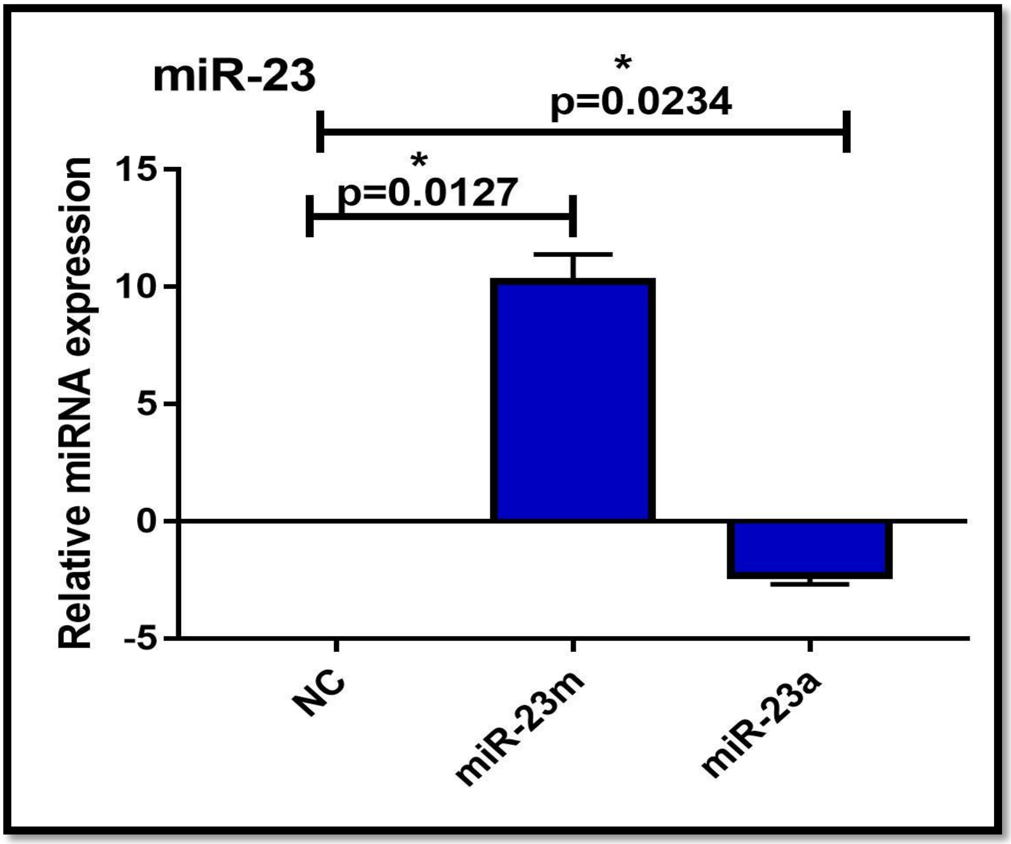

(B)

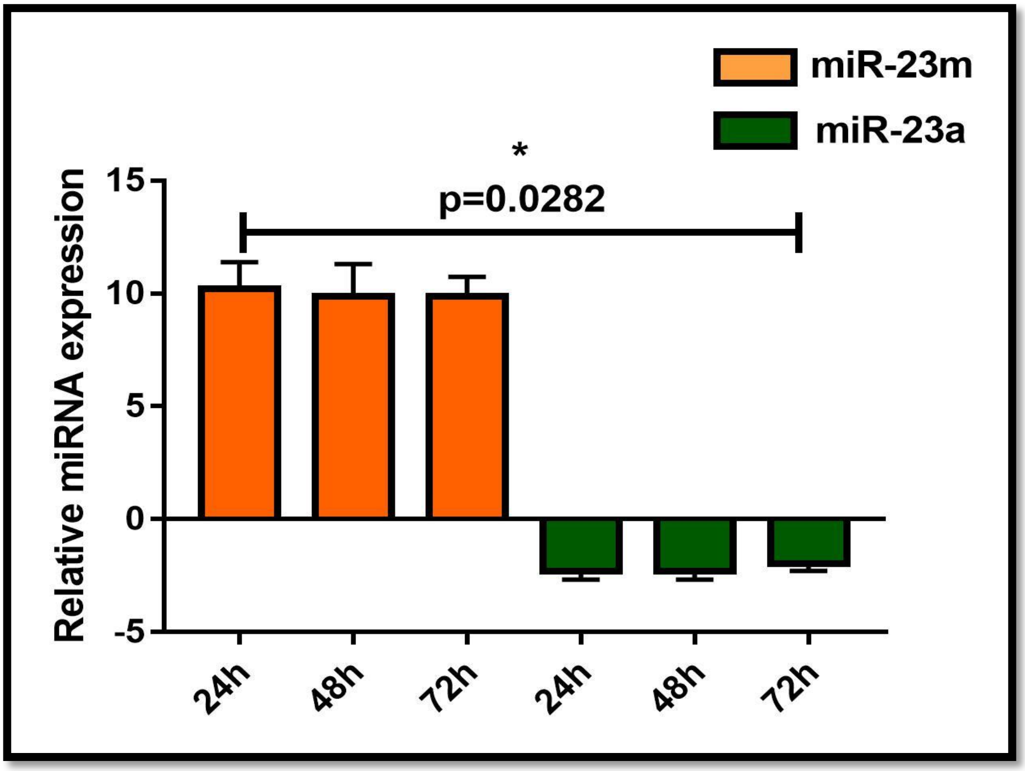

Supp. Figure S10 (A) & (B)

Supp. Figure S1: **(A)** HepG2 cells characterization by morphological analysis **(B)** Dose Response Curve (Fold resistance= 2.3) of HepG2 parental cells and HepG2 sorafenib resistant cells

Supp. Figure S2: Real time graph showing expression analysis of Autophagy genes (***ATG7, Beclin1, LC3II and ATG12)***

Supp. Figure S3: Expression analysis of *c-myc* (A*)* in parental and sorafenib resistant HepG2 cells [R; sorafenib resistant HepG2 cells; (B) Sof2=2 µM sorafenib; Sof4=4 µM sorafenib]

Supp. Figure S4: *In-silico* analysis using miRDB database identifying miRNAs as target of *ATG12* (Autophagy)

Supp. Figure S5: *In-silico* analysis using miRDB database identifying miRNAs as target of *GLS* (Glutamine addiction)

Supp. Figure S6: *In-silico* analysis using miRwalk database identifying miRNAs as target of *ATG12* (Autophagy)

Supp. Figure S7: *In-silico* analysis using miRwalk database identifying miRNAs as target of *GLS* (Glutamine addiction)

Supp. Figure S8: Representation of miR-23b-3p (miR-23) transfection assay in HepG2 cells

Supp. Figure S9: MTT assay showing cell viability in presence of miR-23b-3p mimic **(M)** in nM [10 **(M); 20 (M); 30 (M)]** and miR-23b-3p antimiR **(A) [10 (A); 20 (A); 30 (A)]** in HepG2 parental cells and sorafenib resistant HepG2 cells; nM; nanomolars

Supp. Figure S10: **(A)** Expression analysis of miR-23b-3p (miR-23) with transfection efficiency of miR-23b-3p mimic (miR-23m) and miR-23b-3p antimiR (miR-23a) **(B)** Transfection stability of mimic (miR-23m) and antimiR (miR-23a) at different time periods in parental HepG2 cells
